# Supplementary material for: OsSRF8 interacts with OsINP1 and OsDAF1 to regulate pollen aperture formation in rice
Source: Nat Commun. 2024 May 27;15:4512. doi: 10.1038/s41467-024-48813-0 (PMC11130342; doi:10.1038/s41467-024-48813-0)
Supplement: Supplementary file 1 — Supplementary information [file 41467_2024_48813_MOESM1_ESM.pdf]

OsSRF8 interacts with OsINP1 and OsDAF1 to regulate pollen aperture formation in rice

Chen *et al.*

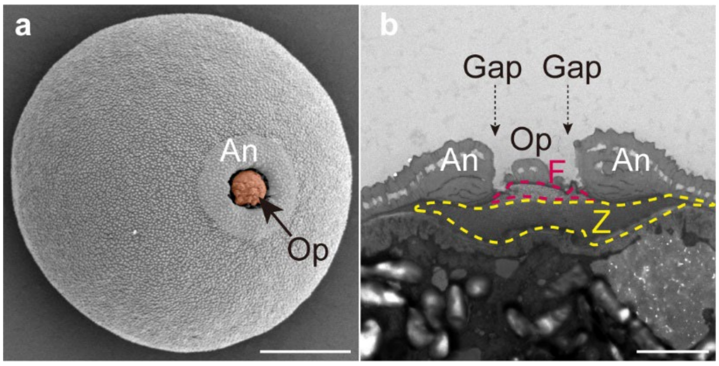

Supplementary Fig. 1 | Observation of pollen aperture for rice. a The surface of rice pollen grain was observed with a scanning electron microscope. The raised annulus is painted in grey; operculum is painted in brown; the space between the annulus and the operculum is represented by the Gap. **b** Transmission electron microscopy observation at the aperture. The complex aperture structure consists of annulus, operculum, and Fibrillar-granular layer and Zwischenkörper layer beneath the whole aperture. There is no exine deposition at all in the Gap area and the The Fibrillar-granular layer and Zwischenkörper layer bridge the annulus and the operculum, effectively sealing the pollen grains. An, annulus; Op, operculum; F, Fibrillar-granular layer; Z, Zwischenkörper layer. ≥10 pollen grains were imaged, with similar results. Scale bars, 10 μm in **a** and 2 μm in **b**.

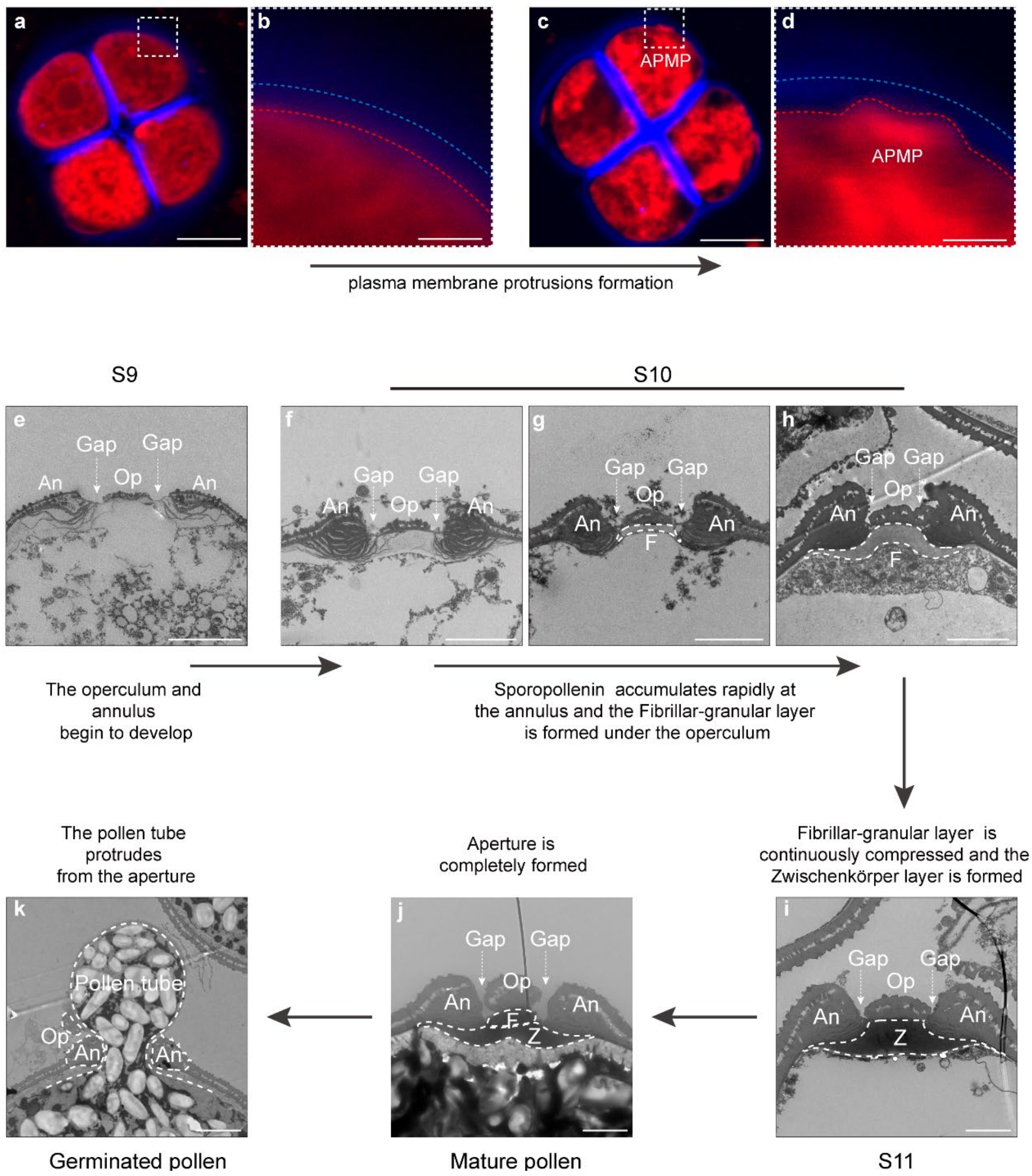

Supplementary Fig. 2 | Developmental process of rice pollen aperture formation. **a-d** Plasma membrane polarization and the formation of APMP. APMP forms on the surface of tetrad microspores. APMP is marked with the dashed square. (**b**) and (**d**) represent enlarged views of the dashed square, respectively. The red dotted lines indicate the boundary of the plasma membrane and the blue dotted lines indicate the outer boundary of the callose wall of microspore at the tetrad stage. Scale bars, 10 µm in (**a**, **c**), 2µm in (**b**, **d**). **e-k** The decoration of aperture. When the tetrad microspores are separated, the annulus and operculum begin to develop. The Gap (no exine deposition) between the annulus and the operculum has become clearly visible (**e**). The

sporopollenin aggregates at the annulus, and the continuously enlarged vacuole concentrates the cytoplasm while compressing the Fibrillar-granular layer (**f-h**). Fibrillar-granular layer is continuously compressed and the Zwischenkörper layer is formed (**i**). The aperture is formed completely (**j**). The pollen tube protrudes from the aperture (**k**). An, annulus; Op, operculum; F, Fibrillar-granular layer; Z, Zwischenkörper layer. Scale bars, 2 μm in (**e-j**), 4 μm in (**k**). These phenotypes were observed three times independently, with similar results.

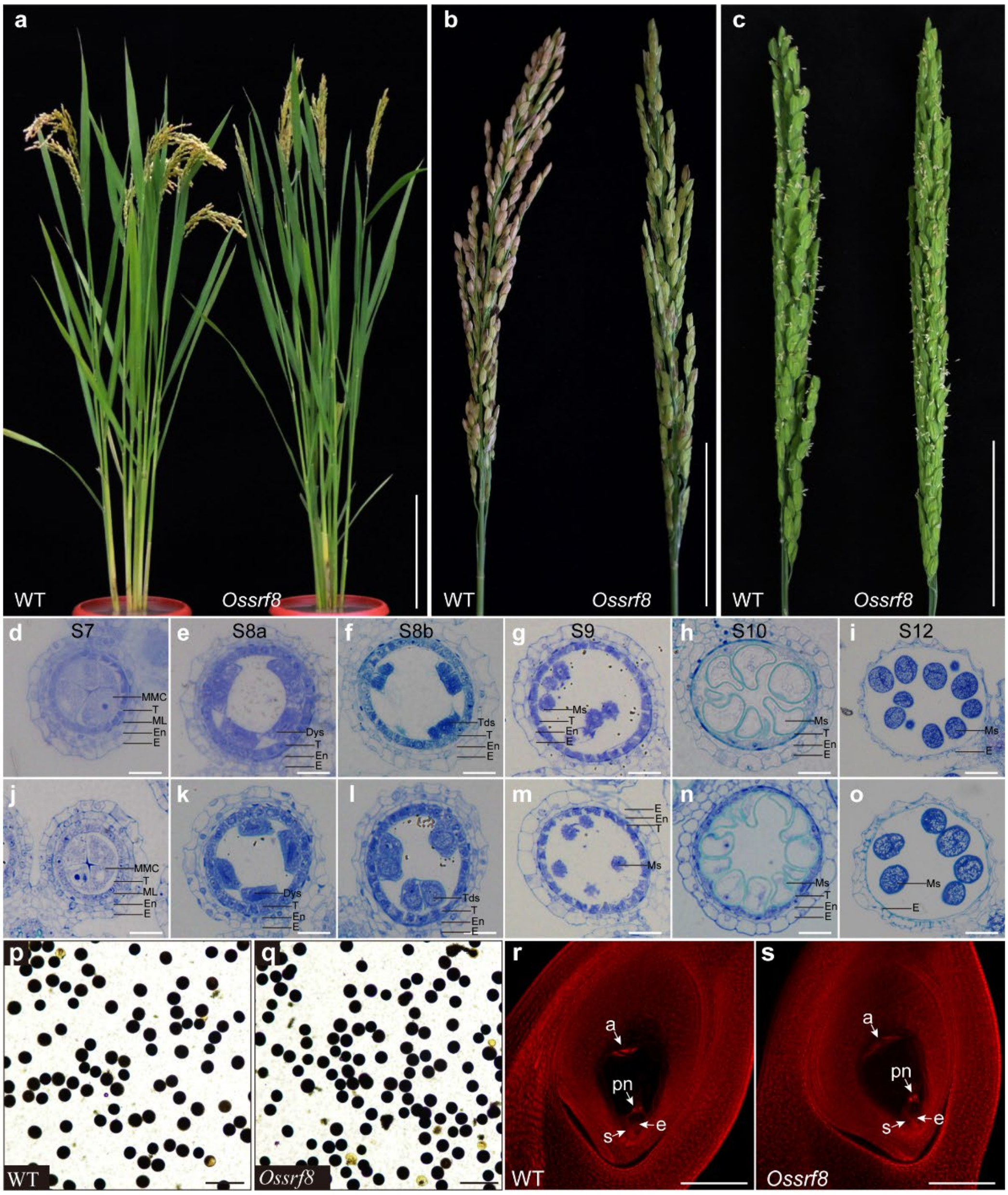

Supplementary Fig. 3 | Phenotypic and cytological Observations of the WT and the *Ossrf8* plants. a Plant morphology of WT and the *Ossrf8* mutant grown in the same environment. Scale bars, 20 cm. **b** Spikelets after seed-setting. Scale bars, 5 cm. **c**, Spikelets after flowering. Scale bars, 5 cm. **d-o** Semi-thin section comparison of different stages of anther development between WT and *Ossrf8* mutant. S7, microspore mother cells stage (**d**, **j**); S8a, dyads stage (**e**, **k**); S8b, tetrads stage (**f**, **l**); S9, free microspore stage (**g**, **m**); S10, vacuolated microspore stage (**h**, **n**); S12, mature pollen stage (**i**, **o**). E, epidermis; En, endothecium; M, middle layer; T, tapetum; MMC, microspore mother cell; Tds, tetrads; Ms, microspore. Scale bars, 20 µm. **p**, **q** Pollen

grains were stained using 1% I_2-KI solution. Scale bars, 100 μm. **r**, **s** Mature embryo sac of the WT and the *Ossrf8*. a, antipode; pn, polar nuclei; e, egg cell; s, synergid. Scale bars, 100 μm. These phenotypes were observed three times independently, with similar results.

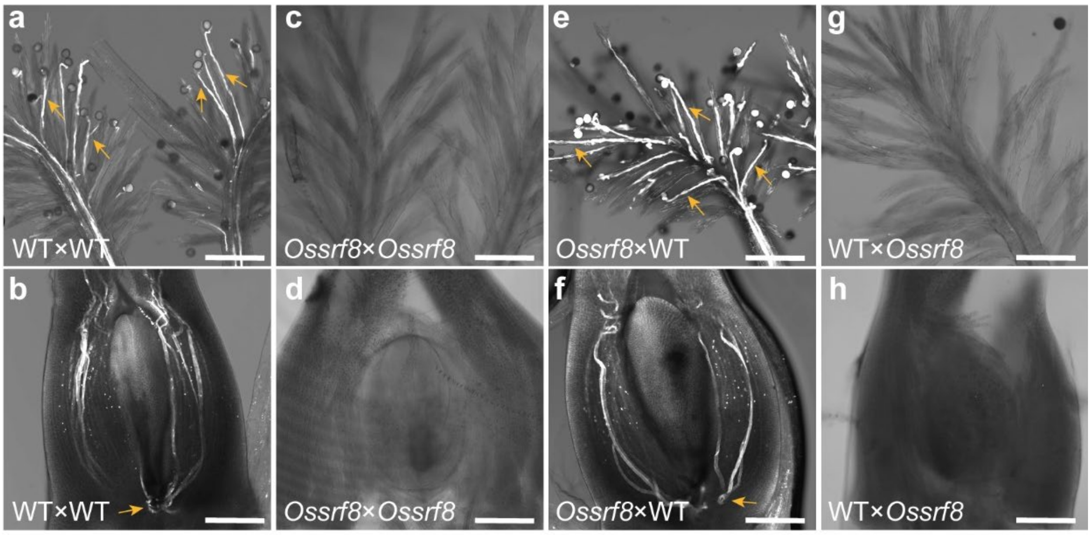

Supplementary Fig. 4 | Observation of in vivo germination of *Ossrf8* mutant and wild-type pollen. a, **b** WT self-pollinated. **c**, **d** *Ossrf8* mutant self-pollinated. **e**, **f** *Ossrf8* mutant as the maternal and the WT as the paternal for saturation pollination experiments. **g**, **h** WT as the maternal and the *Ossrf8* mutant as the paternal for saturation pollination experiments. The orange arrows indicate pollen tubes that grow into the pistils. Phenotypes in **a**-**h** were observed at least three times independently with similar results. Scale bars, 200 μm.

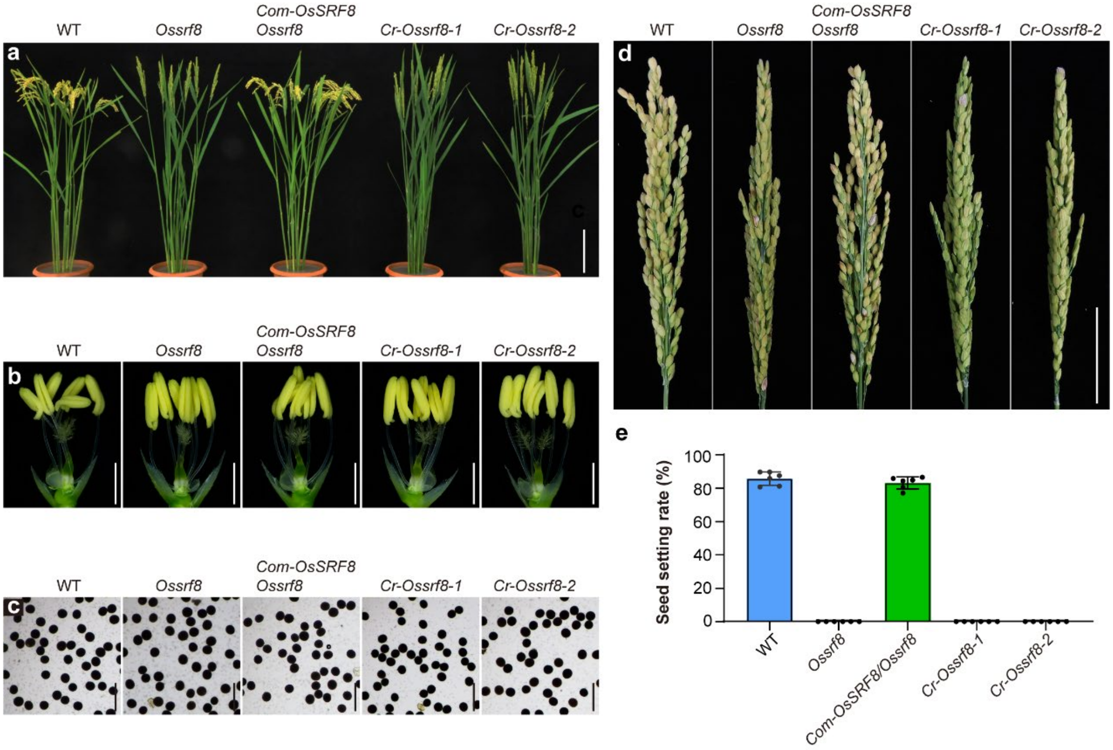

Supplementary Fig. 5 | Phenotypes of the *OsSRF8* knockout and complemented plants. a-d Morphological phenotypes of WT, *Ossrf8*, *Com-OsSRF8/Ossrf8*, *Cr-Ossrf8-1* and *Cr-Ossrf8-2* plants (**a**), flowers (**b**), mature pollen grains (**c**) and mature panicles (**d**). Phenotypes in **a-d** were observed at least three times independently with similar results. Scale bars, 20 cm in (**a**), 2 mm in (**b**), 25 µm in (**c**) and 5 cm in (**d**). **e** Statistics of seed setting rate of WT, *Ossrf8*, *Com-OsSRF8/Ossrf8*, *Cr-Ossrf8-1* and *Cr-Ossrf8-2* mature panicles. Source data are provided as a Source Data file.

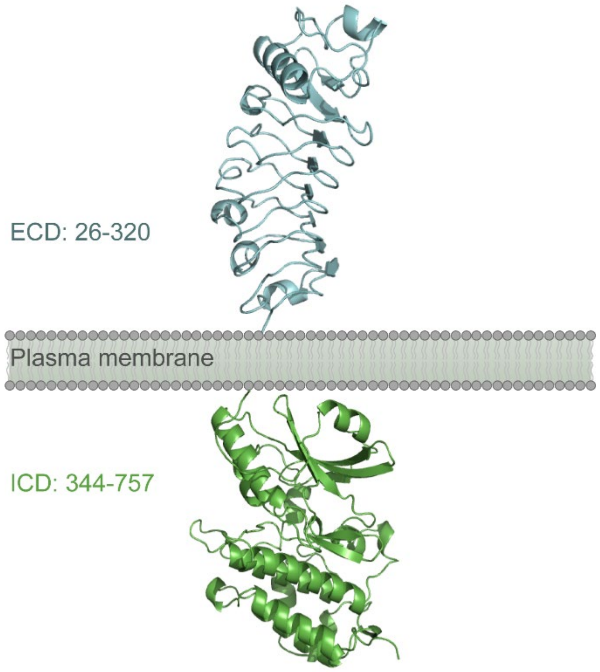

Supplementary Fig. 6 | AlphaFold prediction of OsSRF8 topology. OsSRF8 is predicted to have one predicted transmembrane domain that divides the protein into an Extracellular domain (ECD, aa 26-320), a transmembrane domain (aa 321-343) and an intracellular domain (ICD, aa 344-757). Amino acids 1-25 represent the predicted signal peptide.

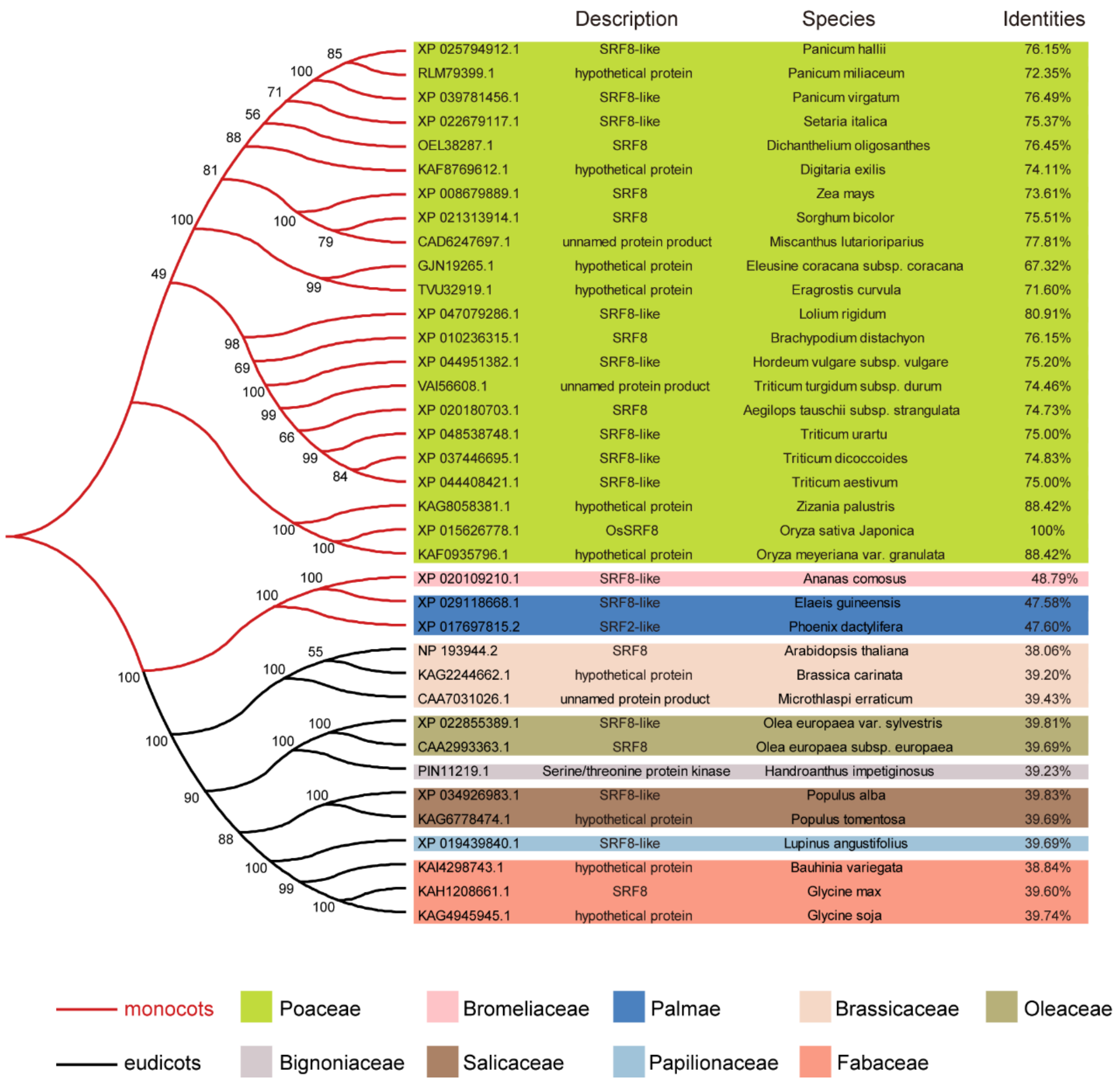

Supplementary Fig. 7 | Evolutionary tree analysis of the OsSRF8 protein. OsSRF8 protein belongs to the class of proteins of STRUBBELIG-RECEPTOR FAMILY (SRF) and is highly conserved within grass species. By homology analysis of the OsSRF8 protein sequence with NCBI BLAST, we obtained several protein sequences that score relatively high in different species. In the Poaceae, the similarity of OsSRF8 is relatively high, all around 70%, while in other species, the similarity is less than 50%. The phylogenetic analysis was performed with MEGA 11 version, basing on the neighbor-joining (NJ), BioNJ and Subtree-Pruning-Regrafting (SPR) algorithm. Bootstrap values are percentage of 1000 replicates.

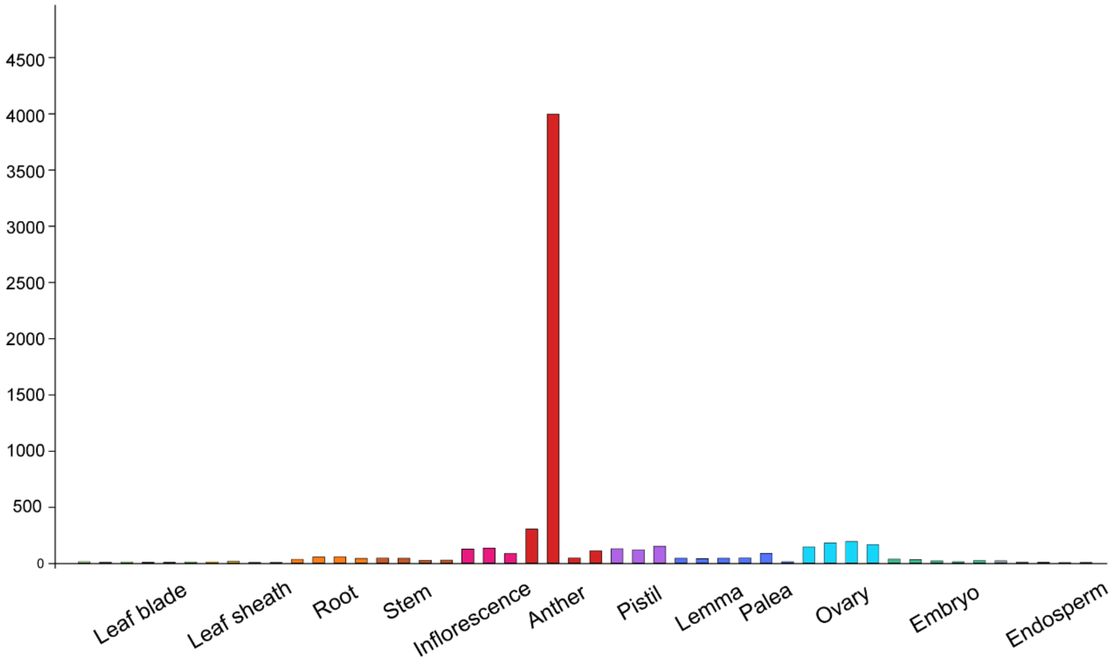

Supplementary Fig. 8 | *OsSRF8* displays highest expression in the early anther. The RNA-Seq data for OsSRF8 were collected from RiceXPro public databases.

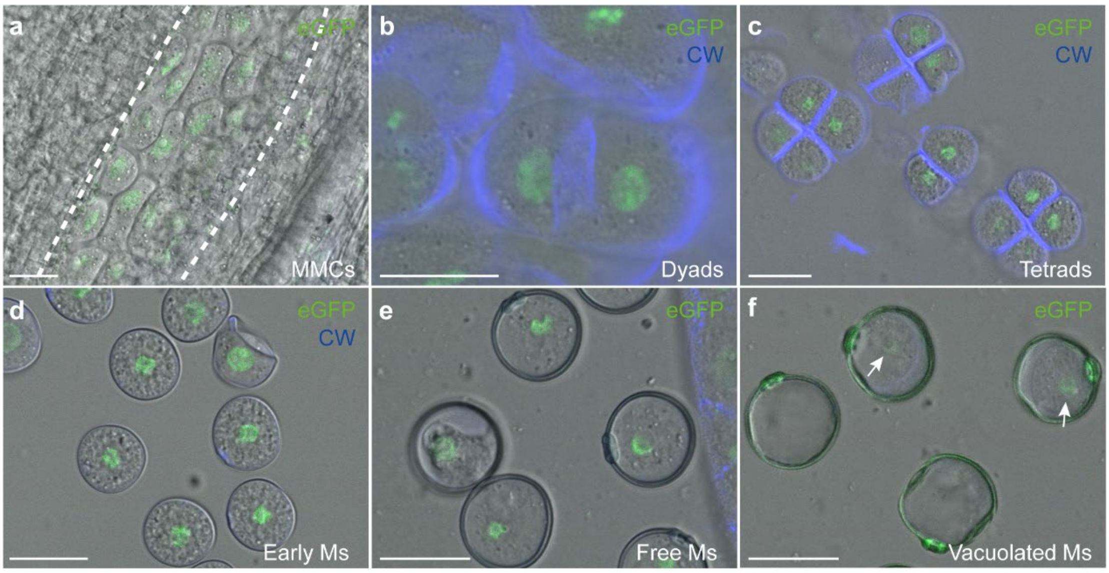

Supplementary Fig. 9 | OsSRF8 is expressed during aperture development. a-f Images of different periods of microspore development expressing the transcriptional fusion construct *OsSRF8pr:H2B-EGFP*. The fluorescence signal of H2B-EGFP is firstly observed in the microspore mother cell (MMCs) and MMCs are demarcated by the white dashed lines (**a**), dyad-stage (**b**), tetrad stage (**c**), early-stage young free microspore (**d**) and free microspore (**e**). However, the signal started to disappear at the vacuolated microspore stage (14 non-fluorescent/total 57 microspores) (**f**). White arrows indicate H2B-EGFP fluorescence signal and the fluorescence signal located at the aperture is derived from autofluorescence. CW, calcofluor white; MMCs, microspore mother cells; Ms, microspores. Five independent T_1 lines were imaged, with similar results. Scale bars, 20 μm.

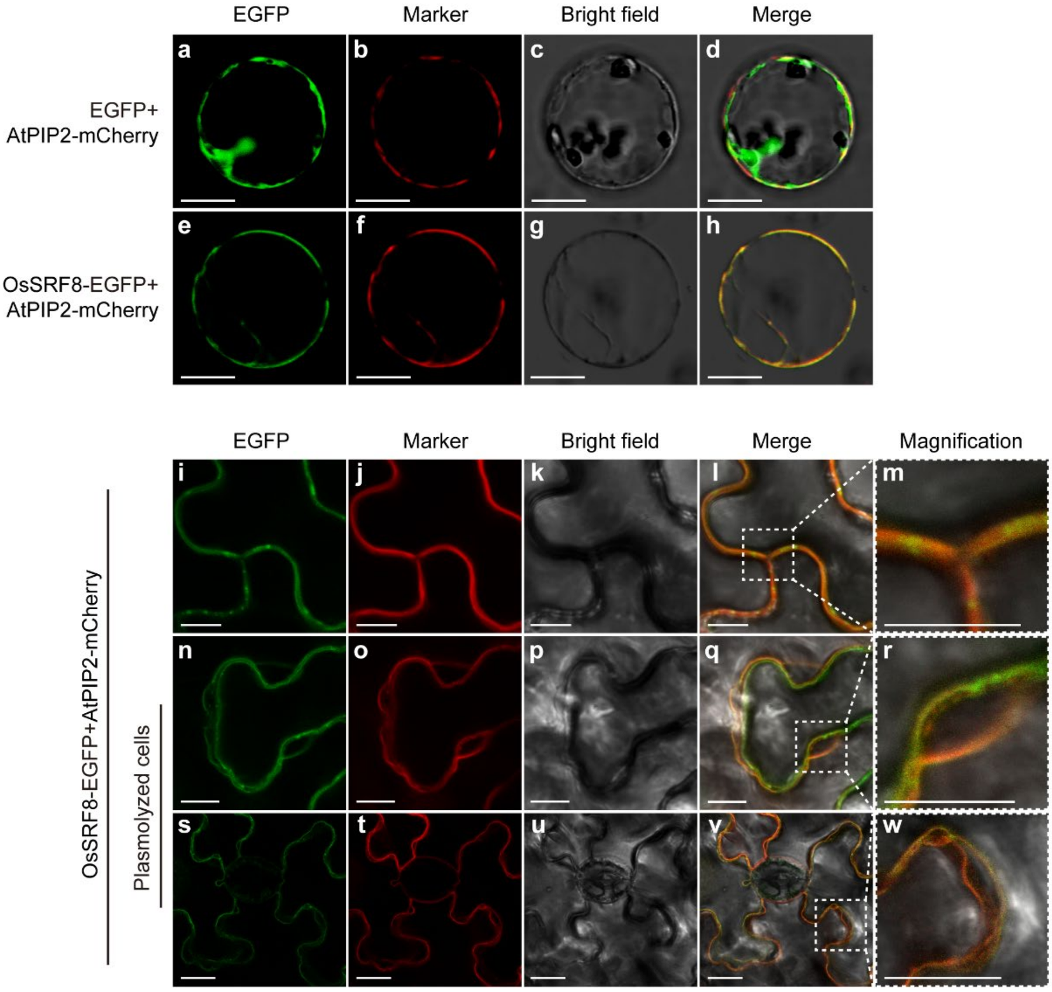

Supplementary Fig. 10 | OsSRF8 is a membrane protein. a-h Representative confocal images of rice protoplasts transiently expressing GFP (**a**) or OsSRF8-EGFP (**e**), the PM marker AtPIP2-mCherry (**b**, **f**), and merged images (**d**, **h**). Scale bars, 15 µm. **i-w** Co-expression of the PM marker AtPIP2-mCherry and the full-length OsSRF8 proteins without Plasmolysis (i-m) and with plasmolysis (n-w) in tobacco epidermal cells. All experiments were repeated at least three times, with similar results. Scale bars, 15 µm in (a-h), 10 µm in (**i-r**) and 20 µm in (**s-w**).

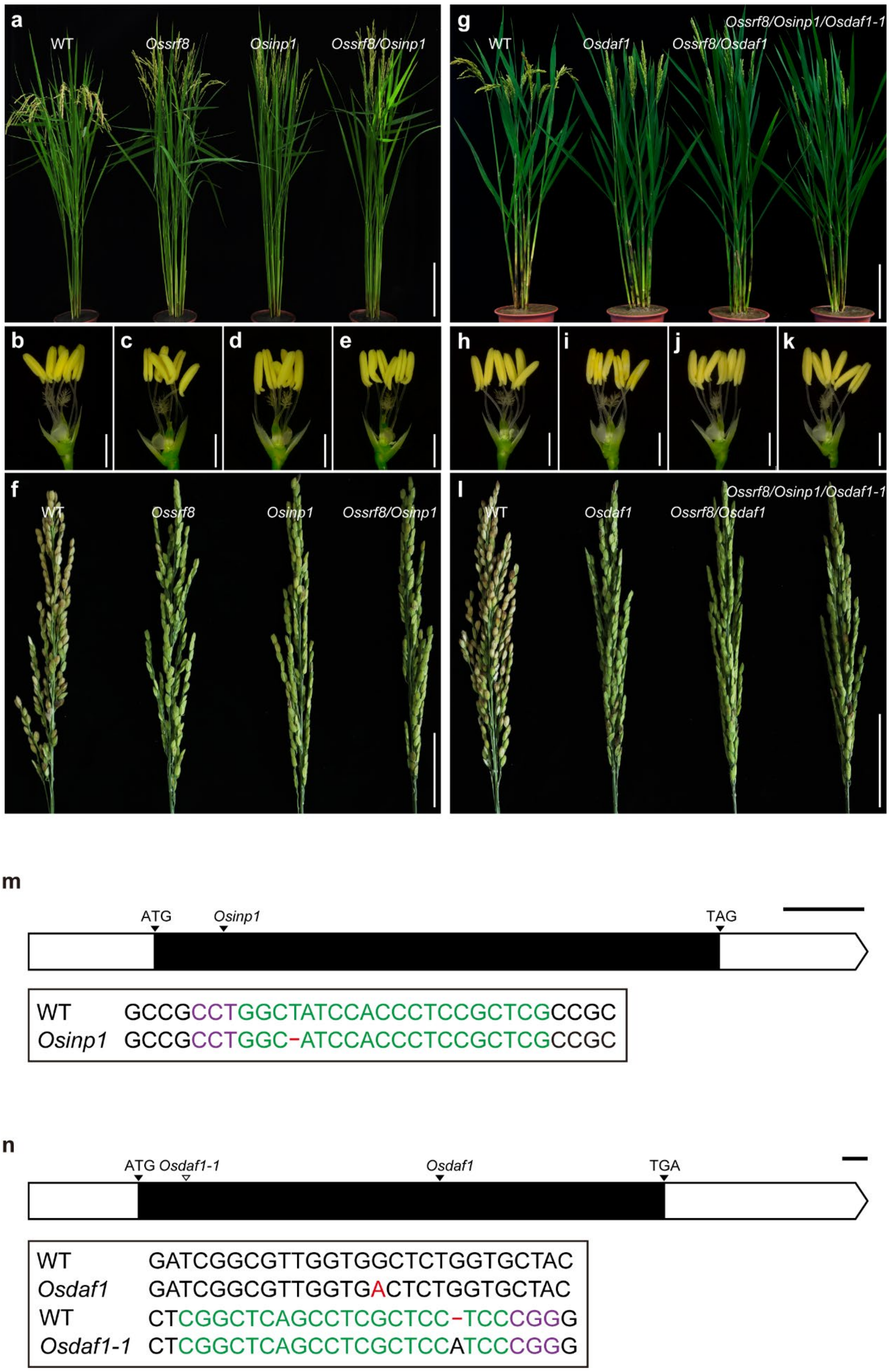
a
WT
Ossrf8
Osinp1
Ossrf8/Osinp1
g
WT
Osdaf1
Ossrf8/Osdaf1
Ossrf8/Osinp1/Osdaf1-1
b
c
d
e
h
i
j
k
f
WT
Ossrf8
Osinp1
Ossrf8/Osinp1
l
WT
Osdaf1
Ossrf8/Osdaf1
Ossrf8/Osinp1/Osdaf1-1
m
ATG
Osinp1
TAG
WT GCCGCCTGGCTATCCACCCTCCGCTCGCCGC
Osinp1 GCCGCCTGGC-ATCCACCCTCCGCTCGCCGC
n
ATG
Osdaf1-1
Osdaf1
TGA
WT GATCGGCGTTGGTGGCTCTGGTGCTAC
Osdaf1 GATCGGCGTTGGTGACTCTGGTGCTAC
WT CTCGGCTCAGCCTCGCTCC-TCCCGGG
Osdaf1-1 CTCGGCTCAGCCTCGCTCCATCCCGGG

106

Supplementary Fig. 11 | Phenotypes of different mutants. a-**f** Comparisons of WT, *Ossrf8*, *Osinp1* and *Ossrf8/Osinp1* plant phenotypes (**a**), flowers (**b-e**), spikelet fertility (**f**). Scale bars, 20 cm in (**a**), 2 mm in (**b-e**) and 5 cm in (**f**). **g-l** Comparisons of WT, *Osdaf1* mutant, *Ossrf8/Osdaf1* double mutant and *Ossrf8/Osinp1/Osdaf1-1* triple mutant plant phenotypes (**g**), flowers (**h**-**k**), spikelet fertility (**l**). Scale bars, 20 cm in (**g**), 2 mm in (**h**-**k**) and 5 cm in (**l**). The phenotypes in **a-l** were observed at least three times independently with similar results. **m**, **n** Display of *OsINP1* (**m**) and *OsDAF1* (**n**) simplified gene structure. The base sequence labeled by the green color represents the knockout target. Scale bars, 100 bases.

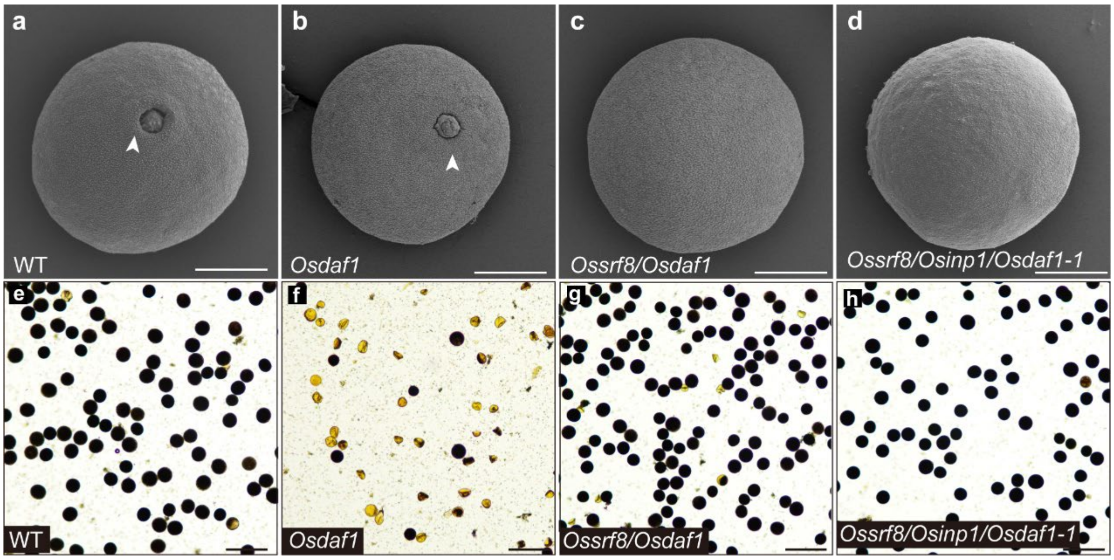

Supplementary Fig. 12 | *Ossrf8* is epistatic to *Osdaf1*. a-d Scanning electron microscopy observation of WT (**a**), *Osdaf1* mutant (**b**), *Ossrf8/Osdaf1* double mutant (**c**), *Ossrf8/Osinp1/Osdaf1-1* triple mutant (**d**) mature pollen grains. White arrow indicates annulus. Scale bars, 10 μm. **e-h** I_2-KI staining observation of WT (**e**), *Osdaf1* mutant (**f**), *Ossrf8/Osdaf1* double mutant (**g**), *Ossrf8/Osinp1/Osdaf1-1* triple mutant (**h**) mature pollen grains. The phenotypes were observed at least three times independently with similar results. Scale bars, 10 μm in (**a-d**) and 100 μm in (**e-h**). A small amount of *Osdaf1* mutant pollen starch filling is normal, but the pollen starch filling returns to normal in the *Ossrf8/Osdaf1* double mutant. The *Osdaf1* mutant does not have the annulus structure, while the double and triple mutants do not have the aperture structure.

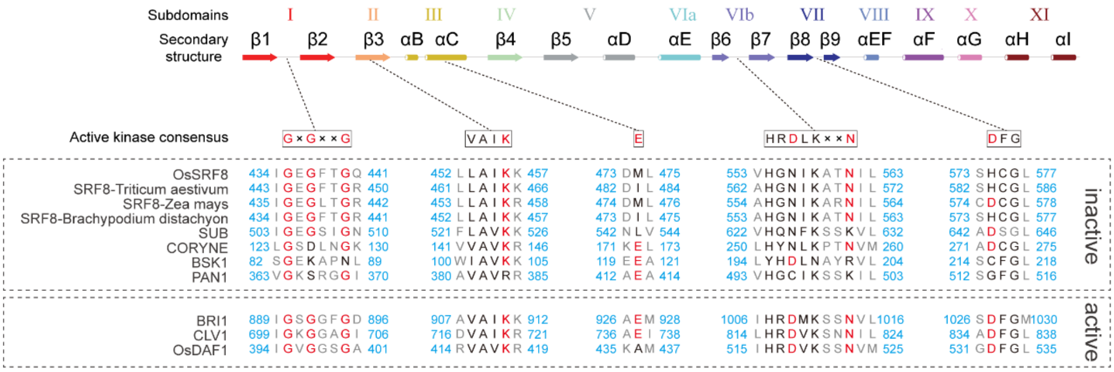

Supplementary Fig. 13 | OsSRF8 protein is predicted as an inactive kinase. Alignment of conventional protein kinase P-loop, VAIK, E, HRD and DFG motifs with their counterparts including homologous proteins of OsSRF8, SUB/CORYNE/BSK1/PAN1 with inactive kinase, and BRI1/CLV1/OsDAF1 with active kinase. In the kinase domain, conserved amino acid residues that are critical for kinase activity are marked red. In the OsSRF8 protein, the Glu residue derived from the C-helix is replaced by Met, the Asp residue derived from the HRD motif is replaced by the polar uncharged amino acid Asn, and the Asp residue derived from the DFG motif is changed to His, all of which imply that the OsSRF8 protein is an inactive kinase.

Supplemental Table 1. Phenotypic and genotypic statistics of *OsSRF8* transgenic complementary plants.

Generation	Transgenic line	Positive			Negative		
		Mutant background		Wild-type background	Mutant background		Wild-type background
		Normal pollen aperture	Defective pollen aperture	Normal pollen aperture	Normal pollen aperture	Defective pollen aperture	Normal pollen aperture
T_0		11	1	47	0	3	7
T_1	#1	14	0	24	0	0	2
	#2	13	1	16	0	0	0
	#3	39	0	0	0	1	0
	#4	18	0	0	0	2	0

Positive and Negative indicate the positive and negative transgenic plants, respectively.

Supplemental Table 2. Phenotypic statistics of pollen aperture for *Ossrf8* and *Osinp1* knockout plants.

Generation	Knockout target	Transgenic line	Number of plants		
			Normal pollen aperture	No pollen aperture	Total
T_0	OsSRF8-Target1		19	7	26
	OsSRF8-Target2		26	15	41
T_1	OsSRF8-Target1	#1	27	13	40
	OsSRF8-Target1	#2	13	7	20
	OsSRF8-Target1	#3	15	4	19
	OsSRF8-Target2	#1	18	10	28
	OsSRF8-Target2	#2	20	8	28
	OsSRF8-Target2	#3	21	7	28
T_0	OsINP1-Target		15	9	24
T_1	OsINP1-Target	#1	28	14	42
	OsINP1-Target	#2	14	6	20
	OsINP1-Target	#3	12	5	17
T_2	OsINP1-Target	#1	15	3	18
	OsINP1-Target	#2	13	7	20

Supplemental Table 3. Fluorescence observation and statistics of transgenic plants expressing *pSRF8-H2B-EGFP*.

Generation	Transgenic line	Positive		Negative
		EGFP signal	No EGFP signal	
T_0		36	0	1
T_1	#1	20	0	0
	#2	21	0	0
T_2	#1	18	0	0

Supplemental Table 4. Fluorescence observation and statistics of vacuolated microspores.

	Microspores with fluorescence	Microspores with no fluorescence	Total
The number of microspores	14	43	57

Supplemental Table 5. Observation of OsSRF8 fluorescent proteins and statistics of transgenic plants.

Generation	Transgenic OsSRF8-eYFP line	Positive		Negative
		With eYFP signal	Without eYFP signal	
T_0		41	0	13
T_1	#1	22	0	0
	#2	18	0	0
T_2	#1	18	0	0
	#2	36	0	0

Supplemental Table 6. Phenotypic and genotypic statistics of *Ossrf8/Osinp1* double mutant plants.

Generation	Number of plants			
	*OsSRF8_/ OsINP1_*	*OsSRF8_/ Osinp1Osinp1*	*Ossrf8Ossrf8/ OsINP1_*	*Ossrf8Ossrf8/ Osinp1Osinp1*
	Normal pollen aperture	No pollen aperture	No pollen aperture	No pollen aperture
F_1	17	0	0	0
F_2	36	8	9	9
F_3	22	8	8	2

Supplemental Table 7. Fluorescence observation and statistics of transgenic plants expressing *OsSRF8-eYFP* in *OsINP1/Osinp1* background.

Generation	*OsINP1/OsINP1*	*OsINP1/Osinp1*	*Osinp1/Osinp1*
F_1	5	4	0
F_2	15	3	3
F_3	7	6	3
	the distribution of OsSRF8-eYFP protein (random or polar)		
F_1	polar	polar	-
F_2	polar	polar	random
F_3	polar	polar	random

Supplemental Table 8. Fluorescence observation and statistics of transgenic plants expressing *OsINP1-EGFP* in *OsSRF8/Ossrf8* background.

Generation	Transgenic line	Positive			Negative
		OsSRF8/OsSRF8	*OsSRF8/Ossrf8*	*Ossrf8/Ossrf8*	_/_
T_0		13	20	4	3
T_1	#1	10	5	6	1
	#2	2	6	2	0
	#3	5	8	6	0
T_2		7	19	10	0
		the distribution of OsINP1-EGFP protein (random or polar)			
T_0		polar	polar	polar	-
T_1	#1	polar	polar	polar	-
	#2	polar	polar	polar	-
	#3	polar	polar	polar	-
T_2		polar	polar	polar	-

Supplemental Table 9. Phenotypic and genotypic statistics of *Osdaf1/Ossrf8* double mutant plants.

Generation	Number of plants			
	*OsDAF1_/ OsSRF8_*	*Osdaf1Osdaf1/ OsSRF8_*	*OsDAF1_/ Ossrf8Ossrf8*	*Osdaf1Osdaf1/ Ossrf8Ossrf8*
	Normal pollen aperture	No annulus pollen	No pollen aperture	No pollen aperture
F_1	19	0	0	0
F_2	18	9	9	1
F_3	28	10	8	4

Supplemental Table 10. Phenotypic and genotypic statistics of *Ossrf8/Osinp1/Osdaf1* triple mutant plants.

Generation	Number of plants					
	Normal pollen aperture			Defective pollen aperture		
	*OsSRF8 Ossrf8/<br>OsINP1_/<br>OsDAF1_*	*OsSRF8 Ossrf8/<br>OsINP1 Osinp1/<br>OsDAF1_*	*OsSRF8 Ossrf8/<br>OsINP1 Osinp1/<br>OsDAF1 Osdaf1*	*Ossrf8Ossrf8/<br>_ _/<br>_ _*	*Ossrf8Ossrf8/<br>Osinp1Osinp1/<br>_ _*	*Ossrf8Ossrf8/<br>Osinp1Osinp1/<br>Osdaf1Osdaf1*
T_0	26	10	3	11	9	7
T_1	35	21	12	29	6	2

Supplemental Table 11. Fluorescence observation and statistics of transgenic plants expressing *OsDAF1-eYFP* in *OsSRF8/Ossrf8* background.

Generation Transgenic repeat events		Positive			Negative
		OsSRF8/OsSRF8	*OsSRF8/Ossrf8*	*Ossrf8/Ossrf8*	_/_
T_0	I	21	8	12	5
	II	11	9	7	41
		the distribution of OsDAF1-eYFP protein (random or polar)			
T_0	I	polar	polar	random	-
	II	polar	polar	random	-

Supplemental Table 12. Fluorescence observation and statistics of transgenic plants expressing *OsSRF8-eYFP* in *OsDAF1/Osdaf1* background.

Generation	*OsDAF1/OsDAF1*	*OsDAF1/Osdaf1*	*Osdaf1/Osdaf1*
F_1	0	1	0
F_2	5	7	5
F_3	7	9	2
	the distribution of OsSRF8-eYFP protein (random or polar)		
F_1	-	polar	-
F_2	polar	polar	polar
F_3	polar	polar	polar

Supplemental Table 13. List of all primers used in this study.

Primer name	Primer sequence	Description
2-10-F	CAGATACCCTCGCAAAAAGG	Map-based cloning assay
2-10-R	CGGACCCCAAAGAAAGAAAG	Map-based cloning assay
DY7-F	GTCAGCTGTTGTTGGCAAGA	Map-based cloning assay
DY7-R	TGGTGAAAACTGAGAACAGCT	Map-based cloning assay
DY8-F	TGCTGCTATGTAGGCTAGGC	Map-based cloning assay
DY8-R	TGACCCCGTCATCAAGGTAC	Map-based cloning assay
DY9-F	AAACCGCCACCGATCGAG	Map-based cloning assay
DY9-R	GGGTTCGGATTCGCATTCTG	Map-based cloning assay
DY13-F	GTGAATCATGGTGGCGAGC	Map-based cloning assay
DY13-R	GCCGGAGATCAGAACGAATC	Map-based cloning assay
DY15-F	TGCACAACCTACAACATGCT	Map-based cloning assay
DY15-R	TGCGAGAAACCTGCAGTAGA	Map-based cloning assay
DY-17-F	GCAAGTTCCTACAATAGCATGC	Map-based cloning assay
DY-17-R	ACAACCTTCCTTTTGGTCTTGT	Map-based cloning assay
DY19-F	GGACCTGGAGGAGATCGTG	Map-based cloning assay
DY19-R	CGATAATATCTACGGCCGTGG	Map-based cloning assay
2-103-F	CGCTCTTGTCTTTACTCCCG	Map-based cloning assay
2-103-R	GCTGCTGCTGCTTCTTTTTC	Map-based cloning assay
Crispr-OsSRF8-1-F	GGCAGCCTCGCCTCCTCCTCGCCG	Crispr assay
Crispr-OsSRF8-1-R	AAACCGGCGAGGAGGAGGCGAGGC	Crispr assay
Crispr-OsSRF8-2-F	GGCATGGGAACCAGTTTCAACC	Crispr assay
Crispr-OsSRF8-2-R	AAACGGTTGAAACTGGTTCCCA	Crispr assay
Crispr-OsINP1-F	GGCACGAGCGGAGGGTGGATAGCC	Crispr assay
Crispr-OsINP1-R	AAACGGCTATCCACCCTCCGCTCG	Crispr assay
Crispr-OsDAF1-R	GGCAGTGGCGCTGCATGACCCGGA	Crispr assay
Crispr-OsDAF1-R	AAACTCCGGGTCATGCAGCGCCAC	Crispr assay
Com-gOsSRF8-F	CCGGCGCGCCAAGCTTATCTGAGTCCACCTAACAGACGTT	*OsSRF8* complementation
Com-gOsSRF8-R	GAATTCCCGGGGATCCGTCAACTTCGGGCAGTACCACTAC	*OsSRF8* complementation
KpnI-eYFP-F	TGTTACTTCTGCACTAGGTACCATGGTGAGCAAGGGCGAGGAGCTG	*OsSRF8* complementation
KpnI-eYFP-R	GAAGGGCTTTAAGATCTGTACAGCTCGTCCAT	*OsSRF8* complementation
3UTR-F	GATCTTAAAGCCCTTCCCTCCACCCCCCCAAT	*OsSRF8* complementation
3UTR-R	GAATTCCCGGGGATCCGTCAACTTCGGGCAGTACCACTAC	*OsSRF8* complementation

pOsSRF8-H2B-EGFP-F	CCGGCGCGCCAAGCTTATCTGAGTCCACCTAACAGACGTT	*OsSRF8* expression pattern
pOsSRF8-H2B-EGFP-R	GAATTCCCGGGGATCCTTAAGATCTGTACAGCTCGTCCAT	*OsSRF8* expression pattern
OsSRF8cds-eYFP-F	GCCCAGATCAACTAGTATGGCGGCGGCGGCGCTGCCTCGC	Subcellular localizations
OsSRF8cds-eYFP-R	TGCTCACCATGGATCCTCTCTGGGAGATGCAGCCTCCGGT	Subcellular localizations
gINP1-EGFP-F	CCAGCCCATAACTCTCCTACATCACTCGCAGTCGAGGCCC	OsINP1 localization
gINP1-EGFP-R	TGTTTGAACGCTGCAGTTACTTGTACAGCTCGTCCATGCC	OsINP1 localization
gDAF1-eYFP-F	CCGGCGCGCCAAGCTTTAGGTGGTGTCTGCGTAGGACGGA	OsDAF1 localization
gDAF1-eYFP-R	GAATTCCCGGGGATCCCTTTGAAAACAAGGTATACACATT	OsDAF1 localization
BD-OsSRF8 (N)-F	CATGGAGGCCGAATTCATGTTCACCGACCCCTCCGATGCTTTA	Y2H assay
BD-OsSRF8 (N)-R	GCAGGTCGACGGATCCTTAGTCGAGAGTGTGACTTCCTGTGCTG	Y2H assay
AD-OsINP1-F	GGAGGCCAGTGAATTCATGCCGAGGCCTCCTCCTCCTCCT	Y2H assay
AD-OsINP1-R	CGAGCTCGATGGATCCTTAACTACCGGGAGAGGCGCGGCA	Y2H assay
AD-OsSRF8 (C)-F	GGAGGCCAGTGAATTCAAAAGCTGCACATACAGTCCTAAG	Y2H assay
AD-OsSRF8 (C)-R	CGAGCTCGATGGATCCTTATCTCTGGGAGATGCAGCCTCCGGT	Y2H assay
BD-OsDAF1(C)-F	CATGGAGGCCGAATTCATGGGCCGGTCAGTGCGCCGCAAAAAT	Y2H assay
BD-OsDAF1(C)-R	GCAGGTCGACGGATCCTTAGCGACAACTGAAGAATGCAGTATT	Y2H assay
OsSRF8 (N)-nLUC-F	GGTACCCGGGATCCATGTTCACCGACCCCTCCGATGCTTTA	LUC assay
OsSRF8 (N)-nLUC-R	GAGATCTGGTCGACTCGAGAGTGTGACTTCCTGTGCTG	LUC assay
cLUC-OsSRF8 (N)-F	CCCGGGGCGGTACCATGTTCACCGACCCCTCCGATGCTTTA	LUC assay
cLUC-OsSRF8 (N)-R	CTCTGCAGGTCGACTCGAGAGTGTGACTTCCTGTGCTG	LUC assay
OsINP1-nLUC-F	GGTACCCGGGATCCATGCCGAGGCCTCCTCCTCCTCCT	LUC assay
OsINP1-nLUC-R	GAGATCTGGTCGACACTACCGGGAGAGGCGCGGCACTG	LUC assay
cLUC-OsINP1-F	CCCGGGGCGGTACCATGCCGAGGCCTCCTCCTCCTCCT	LUC assay
cLUC-OsINP1-R	CTCTGCAGGTCGACCTAACTACCGGGAGAGGCGCGGCA	LUC assay
OsSRF8 (C)-nLUC-F	GGTACCCGGGATCCATGAAAAGCTGCACATACAGTCCT	LUC assay
OsSRF8 (C)-nLUC-R	GAGATCTGGTCGACTCTCTGGGAGATGCAGCCTCCGGT	LUC assay
cLUC- OsSRF8 (C)-F	CCCGGGGCGGTACCATGAAAAGCTGCACATACAGTCCT	LUC assay

cLUC- OsSRF8 (C)-R	CTCTGCAGGTCGACTTATCTCTGGGAGATGCAGCCTCC	LUC assay
OsDAF1(C)-nLUC-F	GGTACCCGGGATCCATGGGCCGGTCAGTGCGCCGCAAA	LUC assay
OsDAF1(C)-nLUC-R	GAGATCTGGTCGACGCGACAACTGAAGAATGCAGTATT	LUC assay
cLUC-OsDAF1(C)-F	CCCGGGGCGGTACCATGGGCCGGTCAGTGCGCCGCAAA	LUC assay
cLUC-OsDAF1(C)-R	CTCTGCAGGTCGACTCAGCGACAACTGAAGAATGCAGT	LUC assay
OsSRF8-nYFP-F	CATTTACGAACGATAGTTAATTAAATGGCGGCGGCGGCGCTG CCTCGC	BiFC assay
OsSRF8-nYFP-R	CACTGCCACCTCCTCCACTAGTTCTCTGGGAGATGCAGCCTC CGGT	BiFC assay
OsINP1-cYFP-F	CATTTACGAACGATAGTTAATTAAATGCCGAGGCCTCCTCCTC CTCCT	BiFC assay
OsINP1-cYFP-R	CACTGCCACCTCCTCCACTAGTACTACCGGGAGAGGCGCGG CACTG	BiFC assay
OsSRF8 (C)-nYFP-F	CATTTACGAACGATAGTTAATTAAATGAAAAGCTGCACATAC AGTCCT	BiFC assay
OsSRF8 (C)-nYFP-R	CACTGCCACCTCCTCCACTAGTTCTCTGGGAGATGCAGCCTC CGGT	BiFC assay
OsDAF1(C)-cYFP-F	CATTTACGAACGATAGTTAATTAAATGGGCCGGTCAGTGCGC CGCAAAAAT	BiFC assay
OsDAF1(C)-cYFP-R	CACTGCCACCTCCTCCACTAGTGCGACAACTGAAGAATGCA GTATT	BiFC assay
35S:OsSRF8 (N)-Flag-F	AGAACACGGGGGACTCTAGAATGTTCACCGACCCCTCCGAT GCTTTA	Co-IP assay
35S:OsSRF8 (N)-Flag-R	CCGTCATGGTCTTTGTAGTCGTCGAGAGTGTGACTTCCTGTG CTG	Co-IP assay
35S:OsINP1-EGFP-F	AGAACACGGGGGACTCTAGAATGCCGAGGCCTCCTCCTCCT CCTCC	Co-IP assay
35S:OsINP1-EGFP-R	CCTCGCCCTTGCTCACACTACCGGGAGAGGCGCGGCACTG	Co-IP assay
35S:OsDAF1(C)-Flag-F	AGAACACGGGGGACTCTAGAATGGGCCGGTCAGTGCGCCGC AAAAAT	Co-IP assay
35S:OsDAF1(C)-Flag-R	CCGTCATGGTCTTTGTAGTCGCGACAACTGAAGAATGCAGTA TT	Co-IP assay
35S:OsSRF8 (C)-EGFP-F	AGAACACGGGGGACTCTAGAATGAAAAGCTGCACATACAGT CCTAAG	Co-IP assay
35S:OsSRF8 (C)-EGFP-R	CCTCGCCCTTGCTCACTCTCTGGGAGATGCAGCCTCCGGT	Co-IP assay
35S:OsSRF8-HA-F	CGGAGCTAGCTCTAGAATGGCGGCGGCGGCGCTGCCTCGC	Co-IP assay

35S:OsSRF8-HA-R	TACATGGATCCCCCGGGTCTCTGGGAGATGCAGCCTCCGGT	Co-IP assay
35S:OsDAF1-Flag-F	CGGAGCTAGCTCTAGAATGCCTCCACGCTGTAGGCGCCTC	Co-IP assay
35S:OsDAF1-Flag-R	TCTTTGTAGTCCATGGATCCGCGACAACTGAAGAATGCAGTATT	Co-IP assay
